# Skin-Derived Precursor Cells as an In Vitro Modelling Tool for the Study of Type 1 Neurofibromatosis

**DOI:** 10.1155/2012/646725

**Published:** 2012-04-01

**Authors:** Araika Gutiérrez-Rivera, Haizea Iribar, Anna Tuneu, Ander Izeta

**Affiliations:** ^1^Tissue Engineering Lab, Bioengineering Area, Instituto Biodonostia, Hospital Universitario Donostia, 20014 San Sebastián, Spain; ^2^Department of Dermatology, Hospital Universitario Donostia, 20014 San Sebastián, Spain

## Abstract

The most characteristic feature of neurofibromatosis type 1 (NF1) is the development of neurofibromas. It has been suggested that these tumors are caused by somatic inactivation of the wild-type *NF1* allele, but the cell that originally suffers this mutation remains controversial. Several lines of evidence support the clonal origin of these tumors, and it has been recently suggested that skin-derived precursor cells (SKPs) could be the cell of origin of dermal neurofibromas. Nullizygous (*NF1^−/−^*) SKPs do give rise to neurofibromas when transplanted to heterozygous mice. Moreover, a nullizygous population of cells that is S100**β** negative is present in human neurofibromas, and *NF1^+/−^* multipotent progenitor cells are seemingly recruited to the tumor. This evidence supports the neurofibroma stem cell hypothesis and a putative involvement of SKPs in the aetiopathogenesis of the disease, suggesting that SKPs could become a valuable tool for the in vitro study of NF1.

## 1. Introduction

The tumor predisposition disorder von Recklinghausen's neurofibromatosis type I (NF1) is one of the most common genetic disorders of the nervous system, affecting 1 in 3500 individuals worldwide [1–4]. The disease is caused by mutation in the *NF1* gene (located on chromosome 17q11.2) that encodes the tumor suppressor protein neurofibromin, a GTPase-activating protein (GAP) [5, 6].

Neurofibromas are complex tumors that contain proliferating Schwann-like cells and other local supporting elements of the nerve fibers, as perineurial-like cells, fibroblasts, endothelial cells, pericytes, and vascular smooth muscle cells, as well as infiltration of mast cells [7]. Although several reports have studied which cell originates this tumor, the present data are somewhat contradictory. In this paper we will address the issue of the cell of origin for dermal neurofibromas to explore if the available data support the cancer stem cell hypothesis. We will discuss recent findings in the light of possible involvement of the so-called skin-derived precursor cells in the aetiopathogenesis of this complex disease.

## 2. Skin-Derived Precursors (SKPs)

Skin-derived precursors (SKPs) are a population of neural crest-derived multipotent precursor cells present in both human and mouse dermis. They can be identified in vitro as nonadherent cells isolated from the dermis that proliferate and self-renew in response to growth factors FGF-2 and EGF. Under specific differentiation conditions, they give rise to progeny of the neuronal, glial, and mesodermal lineages [8–14].

SKPs thus derive from the dermis and apparently are distinct from mesenchymal stem cells and from central nervous system neural stem cells [8, 13], although they express genes characteristic of embryonic neural crest cells, such as *Slug, Snail, Twist*,* Pax3, *and* Sox9 *[8].

In vitro, SKPs can be differentiated into mesodermal lineages such as SMA+ smooth muscle cells and adipocytes, as well as into neural crest-derived tissues such as neurons and Schwann cells [8, 13]. In particular, SKPs give rise to cells with neuronal morphology that express the pan-neuronal markers *β*III tubulin and neurofilament-M and proteins characteristic of peripheral neurons such as p75NTR, peripherin, NCAM, tyrosine hydroxylase, and dopamine *β*-hydroxylase. SKPs can also be differentiated into bipolar cells coexpressing glial fibrillary acidic protein GFAP, CNPase, S100*β*, and p75NTR, typical markers of cells with a differentiated Schwann phenotype, as well as MBP and P0 peripheral myelin protein [8, 13].

When transplanted in ovo into the chick neural crest migratory stream, SKPs mostly migrated into peripheral neural crest targets such as spinal nerve, dorsal root ganglia, and skin and expressed S100*β* [8]. In vivo, it has recently been reported that SKPs derive from Sox2+ follicle-associated dermal precursors and show characteristics of dermal stem cells. In this respect, they contribute to dermal maintenance, wound healing, and hair follicle morphogenesis [15].

## 3. Type 1 Neurofibromatosis (NF1)

The primary clinical feature of NF1 is the development of benign peripheral nerve sheath tumors, termed neurofibromas [16]. In a small percentage of NF1 patients, a particular type of neurofibromas (plexiform, see below) progress to malignant peripheral nerve sheath tumors (MPNSTs). NF1 patients are also predisposed to astrocytic brain tumors, pheochromocytoma, and juvenile myelomonocytic leukaemia [2, 17]. Noncancerous symptoms of the disease may include intellectual deficits, bone deformations, benign lesions of the iris (Lisch nodules), axillary freckling, and hyperpigmentation defects of the skin known as café-au-lait macules. Because many of the cardinal features of the disease affect neural crest-derived tissues, NF1 is considered as a neurocristopathy [16, 18–20].

NF1 is a dominantly inherited genetic disease. Half of the NF1 patients have inherited their *NF1* mutation and the other half are caused by a de novo *NF1* mutation, suggesting that the *NF1* locus may represent a mutational hotspot in the human genome [3, 4, 16, 21]. Neurofibromin, the *NF1* gene product, has a Ras GTPase activating (RasGAP) activity and negatively regulates Ras signaling [22, 23]. Neurofibromin functions as a tumor suppressor protein expressed in many cells although it is more abundant in cells from the nervous system such as neurons, Schwann cells, astrocytes, and oligodendrocytes as well as in leukocytes [6, 24, 25]. Loss or reduced neurofibromin expression leads to an increased Ras activity and it has been associated with increased mammalian target of rapamycin (mTOR) activity in astrocytes and Schwann cells [26, 27]. Moreover, neurofibromin plays a key role in the generation of cyclic AMP (cAMP) in both neurons and astrocytes [28, 29]. Loss of heterozygosity (LOH) in the inherited wild-type allele has been detected in some tumor types in NF1 patients, although it has been demonstrated that heterozygosity for *NF1* is a key element for the development of many NF1 symptoms, including neurofibroma formation [30]. An additional complexity of the disease is its variable phenotypic expression, suggesting that modifier genes and epigenetic phenomena may play an important role in disease manifestations [2].

## 4. Neurofibroma Subtypes and CellularComponents

The most common and complex feature of NF1 is the development of benign peripheral nerve sheath tumors or neurofibromas. Neurofibromas were classified by WHO into five subtypes [31]: localized cutaneous, localized intraneural, plexiform, diffuse cutaneous, and soft tissue diffuse neurofibromas (elefantiasis neuromatosa).

Cutaneous neurofibromas reside exclusively in the skin and occur in virtually all individuals with NF1. They initially appear at puberty and increase in number with age and during pregnancy, suggesting a hormonal component in disease development [32–35]. These benign tumors, ranging from 0.1 to several cm in diameter, grow as discrete lesions in the dermis. Patients sometimes develop thousands of these tumors. Depending on their location, they can be painful and disfiguring for the patient and thus affect their quality of life. In contrast, plexiform neurofibromas develop internally along the plexus of major peripheral nerves and become quite large, sometimes involving an entire limb or body region [36]. They occur in about 30% of the individuals and are thought to be congenital. While these tumors are also benign, they are debilitating and may progress to malignancy [37, 38]. The cellular make-up of these lesions is generally similar to that of dermal lesions.

**Figure 1:**
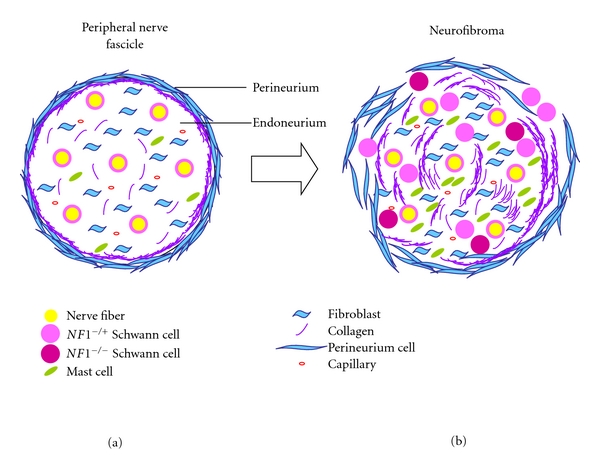
Cellular organization of a normal nerve shaft and a neurofibroma. (a) Nerve shafts are made up of axons and associated Schwann cells. Endoneurium is connective tissue composed by collagen, fibroblasts, mast cells, capillaries and extracellular matrix. Collagen fibers are tighter and more compact near the perineurium. The perineurium is composed by flattened fibroblasts, collagen and elastic fibres. (b) In a neurofibroma, the cells are the same as in a normal nerve shaft, but increased in number. There are more Schwann cells and they can be dissociated from axons. Two kinds of Schwann cells can be detected: *NF1^+/−^* and *NF1^−/−^*. Fibroblasts and mast cells are also increased in number but they are all heterozygous (*NF1^+/−^*). The collagen deposits are also increased and perineurium is usually disrupted.

In a physiological situation, a single peripheral nerve shaft is associated with myelinating or nonmyelinating Schwann cells. Several nerve fibers and associated Schwann cells are clustered into a nerve fascicle, each fascicle being surrounded by concentric layers of perineurial cells. Fibroblasts, endothelial cells, and occasional mast cells are also present in a normal nerve fascicle (Figure 1) [7, 16, 37, 38]. Neurofibromas contain all of the cell types found in normal peripheral nerve but in inappropriate numbers. Moreover, Schwann cells are found dissociated from nerves and the perineurium is often disrupted. Large amounts of intercellular collagen and ground substance are also typically present in neurofibromas [16].

## 5. How Many Mutagenic Events Are Needed for Neurofibromas to Arise?

The penetrance of NF1 is 100% by age 20, although the degree of severity is highly variable, even among family members that present the same mutation [1, 39].

Two types of congenital NF1 mutations have been found to influence neurofibroma number [40–42]. However, these two types of mutations affect only a small percentage of NF1 patients, and, moreover, patients bearing the same germline mutation can exhibit a very different number of dermal neurofibromas [43, 44], indicating that other mechanisms are implicated in neurofibroma formation.

Somatic mutations in the *NF1* gene have been found in tumors associated with NF1, leading to functional loss of both alleles of the gene [45–47]. For example, loss of heterozygosity (LOH) in chromaffin cells initiates pheochromocytomas, and LOH in melanocytes produces pigmented lesions such as café-au-lait macules and Lisch nodules. LOH in myeloid cells induces myelomonocytic leukaemia, and LOH in glial cells permits astrocytoma formation [48–53].

It has also been suggested that neurofibromas are caused by somatic inactivation of the wild-type *NF1* allele, leading to complete functional abrogation of the gene [45, 54, 55]. LOH in Schwann progenitor cells permits plexiform neurofibroma formation [30, 56], and it has been suggested that LOH in skin-derived precursors leads to cutaneous neurofibroma formation [34]. Using both *NF1* intragenic polymorphisms and markers from flanking and more distal regions of chromosome 17, Colman et al. demonstrated loss of heterozygosity (LOH) of the *NF1* gene in eight neurofibromas from 22 patients and Serra et al. found LOH in 15 out of 60 dermal neurofibromas [55, 57]. Moreover, Sawada et al. identified a somatic deletion of the *NF1* gene in a dermal neurofibroma with a defined germline mutation [54]. LOH has also been detected in plexiform neurofibromas [58–60].

One possible explanation for the lack of allele loss detection in some tumors is that a more subtle somatic *NF1* mutation occurred (point mutation, small deletion, insertion, or modification through epigenetic mechanisms). These changes do not produce loss of closely linked polymorphic marker loci [61]. Alternatively, LOH may stay undetected because the presence of normal stromal or inflammatory tissue within the tumors increases sample background. Nevertheless, mechanisms that do not involve inactivation of the normal allele cannot be excluded. In dermal neurofibromas, local trauma can be a factor in the development of the tumors [62] and it has been suggested that dermal neurofibromas could be hyperplastic instead of neoplastic lesions, due to a poorly regulated wound healing in NF1 haploinsufficient tissues [63–65]. However most experts agree that these lesions are true neoplasms and are not hyperplastic.

## 6. Which Neurofibroma Cells Harbor Somatic NF1 Mutations?

Being a complex genetic disease with tumors of multicellular composition, the question arises which cell type within the tumor presents the secondary somatic mutations that characterize the pathological presentation of the dermal neurofibromas. Although *NF1^−/−^* fibroblasts exhibit greater proliferation capacity than their normal and heterozygous counterparts [63, 66], they are not normally found in tumors since only Schwann cells carry a double inactivation of the *NF1* gene [59, 61, 67–69].

Two different populations of S100*β*+ cells (presumably terminally differentiated Schwann cells) have been demonstrated within in vitro cultures obtained from dermal neurofibromas, indicating that both *NF1* Schwann cell subtypes (+/−) and (−/−) coexist in these tumors [61, 68]. This fact may be explained through two alternative possibilities: (i) the second hit mutation occurred as a secondary event within a neurofibroma that had already developed polyclonally, and thus only a subpopulation of S100*β*+ cells is (−/−), or (ii) the tumors arose through a two-hit mechanism within a stem/progenitor cell that gave rise to most tumor cells, but the proliferating neoplastic clone stimulated the proliferation of infiltrating nonneoplastic cells such as heterozygous Schwann cells, mast cells, and fibroblasts.

The influence of a heterozygous environment in plexiform neurofibroma development supports the latter theory. In a conditional plexiform neurofibroma mice model (*NF1* ^flox/−^; Krox20cre), haploinsufficient stromal and mast cells (*NF1^+/−^*) are necessary and limiting for neurofibroma development [30, 70]. Accordingly, *NF1^−/−^* Schwann cell-derived secreted stem cell factor (SCF) causes a hyperactive recruitment of *NF1^+/−^* mast cells [71]. Furthermore, *NF1* mast cells secreted 2.5-fold higher TGF*β* than wt mast cells, leading to a heightened fibroblast proliferation, migration, and collagen production [72]. In all, these data reinforce the idea that heterozygous fibroblast and mast cells may play a key role in the neurofibroma pathogenesis [70].

## 7. Does Neurofibroma Originate from Stem/Progenitor Cells?

Cancer is a heterogeneous disease and tumors present a significant morphological, phenotypic, genetic, kinetic, and functional diversity. Several lines of evidence suggest that this heterogeneity could be due to a hierarchical organization of tumors that resembles normal tissue development. However another possible explanation is that tumor cells are biologically equivalent and that heterogeneity derives from extrinsic or intrinsic influences that result in stochastic responses [73]. Strong evidence points to the importance of stem cells in the initiation and long-term maintenance of several cancers, as malignant germ cell cancers [74, 75], leukemias [76, 77], nervous system [78], breast [79] and colon cancers [80–83]. In these cancer types, several markers have been identified to distinguish the so called “cancer stem cells” that may form tumors when serially transplanted into immunocompromised NOD/SCID mice as compared to nontumorigenic cancer cells that do not present self-renewal capacities. Nevertheless, it is worth noting that in some cancers, most tumor cells fulfill this tumorigenic potential [84–86] and that the NOD/scid mouse transplantation assay sometimes might underestimate the frequency of human cancer cells with tumorigenic potential [85, 86].

The two-hit tumor suppressor hypothesis for NF1 predicts that all cells carry a constitutional mutation and a particular cell acquires a second mutation to initiate tumor formation [87]. Based on the two-hit model of tumorigenesis, tumor cells in neurofibromas should be of clonal origin. Nevertheless, while both alleles are inactivated in NF1-associated malignancies, the clonal nature of the neurofibromas is controversial (see below) [58, 67, 88–91].

Interestingly, there is strong evidence that an adult multipotent stem/progenitor cell could be the cell of origin for cutaneous neurofibromas. It has been demonstrated that plexiform neurofibromas originate from embryonic neural crest-derived progenitors [30, 56, 92–96] and mice that develop plexiform tumors with 100% frequency fail to develop dermal tumors. Moreover, plexiform neurofibromas are congenital while cutaneous neurofibromas arise in puberty. The facts that dermal neurofibromas arise in the adulthood and locate in the dermis suggest the idea that dermal adult progenitor cells could be the source of these tumors [34]. Furthermore, the close relationship observed between the development of cutaneous neurofibromas and hair follicle proximity suggests that adult progenitor cells residing in the hair follicle may be the origin of these tumors. There is evidence that the neurofibromas arise in the hair follicle vicinity and even small neurofibromas can be detected histologically in close contact with the hair follicle, in otherwise apparently healthy skin areas [97, 98]. Mechanical trauma has also been suggested to play a role in the pathogenesis of neurofibromas, that is, some neurofibromas appear to arise as a dysplastic response to crush trauma [99].

Several populations of stem/progenitor cells have been described to reside in the hair follicle or surrounding areas [81, 100–114], some of them being potential candidates for an involvement in NF1 pathogenesis. Recently, it has been speculated that recruitment of Nestin+ multipotent *NF1^+/−^* precursor cells is associated with cutaneous neurofibroma development [97]. Histologically, nestin-positive small blood vessels and spindle-shaped tumor cells can be detected in the neurofibromas. In accordance with this hypothesis, S100*β*−/*NF1*− cells are detected in high proportion (16–31%) in neurofibromas. This fact could indicate the presence of multipotent stem cells that have suffered a second-hit mutation, although a dedifferentiation from S100*β*+/*NF1*− Schwann cells, also present in the tumor, can not be excluded [91].

Finally, there is strong evidence that SKPs could be the cell of origin for dermal neurofibromas [34]. Cre-mediated recombination of *NF*1^lox/−^ SKPs induced in vitro loss of the wt allele in these cells. When transplanted into the same *NF*1^lox/−^ mice that originated these cells, *NF*1^−/−^ SKPs (but not control *NF*1^lox/−^ cells) then initiated dermal neurofibromas. However, tumor formation was only efficient in female recipients that were pregnant at the time of implantation, highlighting the hormone sensitivity observed in NF1 patients and the importance of the microenvironment during neurofibroma formation. Furthermore, deletion of *NF*1 in the skin of CMV-CreERt2 *NF*1^lox/−^ mice after topical application of tamoxifen led to local dermal neurofibroma formation, supporting the notion that the cell of origin for these tumors resides within the skin at close range of topical tamoxifen application [34, 115].

## 8. SKPs as a Tool for In Vitro Modelling of NF1 Features

Several lines of evidence now point to a stem cell origin of dermal neurofibromas. On the one hand, a number of studies have assessed the clonal origin of neurofibromas, based on X chromosome inactivation (XCI) clonality assay. In our view, the results are still controversial since (i) clonal cell origin may not formally be proven through XCI analyses and (ii) studies have generally been performed with low patient numbers. For instance, Skuse et al. studied eight dermal neurofibromas and concluded that all of them were of clonal origin [90]. Tucker et al. also found evidence for clonality in some of the six neurofibromas studied [91], suggesting that although other mechanisms could be at stake, at least in some neurofibromas a unique stem cell may have suffered a second-hit mutation, giving rise to a nullizygous Schwann cell progeny. On the other hand, only one kind of somatic mutation has been found in every neurofibroma analyzed and different neurofibromas of the same patient present different somatic mutations [61, 68], reinforcing the neurofibroma stem cell hypothesis. Moreover, multipotent stem cells (termed neurofibroma-derived precursor cells or NFPs) have been isolated from dermal neurofibromas. These precursors express Nestin and show a multipotent differentiation potential, giving rise to Schwann cells, neurons, epithelial cells, and adipocites [97]. However NFPs do not contain the somatic *NF1* mutation and thus their relationship with NF1 pathogenesis is currently unclear. Similarly, characterization of cells present in neurofibromas by S100*β*, a marker for the Schwann lineage, has demonstrated that a nullizygous population (*NF1^−/−^*) that is negative for S100*β* expression is present in neurofibromas. Although it cannot be discarded that they could be dedifferentiated Schwann cells, it is also possible that they could be progenitor cells that have suffered the somatic mutation and that generate the Schwann cells present in the tumor. In any case, cell characterization by a single marker is less than optimal and too many interpretations of these results are possible as to extract any meaningful conclusion.

**Figure 2:**
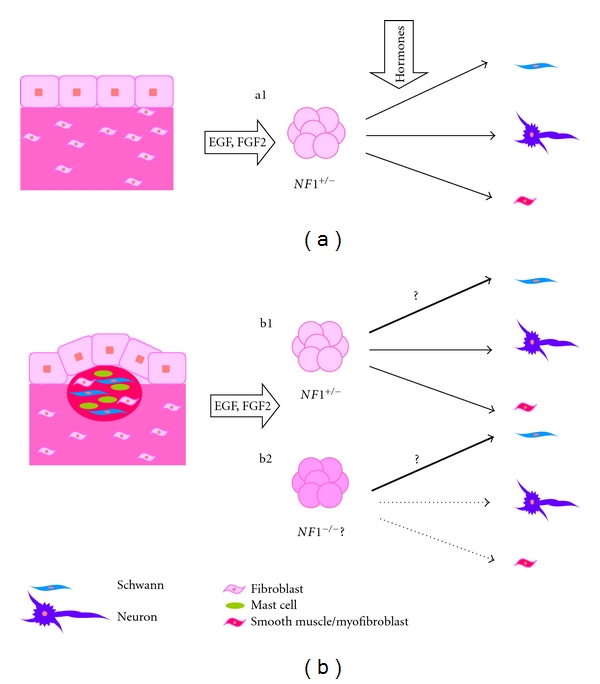
Isolation of SKPs from healthy skin and a neurofibroma of an NF1 patient. (a) Dermal multipotent stem cells form spheres in vitro, in response to EGF and FGF. In NF1 patients, SKPs from healthy skin should give rise to *NF1^+/−^* SKPs in vitro (a1). SKPs can differentiate into glial, neuronal, and mesodermal lineages. If SKPs are isolated from NF1 patient neurofibromas, *NF1^+/−^* SKPs (b1) are expected to form in vitro, under standard culture conditions. If *NF1^−/−^* (b2) SKPs may be isolated has to be determined. If SKPs are the cell of origin of neurofibromas, they might present a predisposition to differentiate into the glial lineage.

Recently, an elegant study showed that *NF1^+/−^* SKPs could form neurofibromas in a conditional mouse model, although a key role for tumor environment was also found [34]. To date there is no data on involvement of SKPs in human neurofibroma development, although *NF1^+/−^* multipotent progenitor cells are supposedly recruited to form dermal neurofibromas [97]. If SKPs were the cells of origin of dermal neurofibromas, *NF1^−/−^* SKPs should be present within NF1 patient neurofibromas, although *NF1^+/−^* SKPs should also be detected. If these putative *NF1^−/−^* SKPs would present a predisposition to differentiate preferentially into the Schwann cell lineage should also be explored (Figure 2).

## 9. Conclusions

In summary, current evidence supports the notion that, at least in murine models, skin-derived precursor cells (SKPs) might be a cell of origin for dermal neurofibromas. It is also conceivable that human SKPs might be the cell of origin of neurofibromas, although formal proof for this is lacking. Isolation of SKPs from human neurofibromas could demonstrate if these dermal multipotent stem cells bear the somatic mutation and whether or not this mutation confers a predisposition to these precursor cells to differentiate into the Schwann cell lineage. Furthermore, isolation of SKPs from healthy skin of NF1 patients could demonstrate if there are SKPs with the somatic mutation, even in areas where the neurofibroma is histologically undetectable. In conclusion, SKPs may become a useful tool for the in vitro study of the neurofibromatosis type 1 syndrome.
